# The Crucial Changes of Sit-to-Stand Phases in Subacute Stroke Survivors Identified by Movement Decomposition Analysis

**DOI:** 10.3389/fneur.2018.00185

**Published:** 2018-03-26

**Authors:** Yu Rong Mao, Xiu Qin Wu, Jiang Li Zhao, Wai Leung Ambrose Lo, Ling Chen, Ming Hui Ding, Zhi Qin Xu, Rui Hao Bian, Dong Feng Huang, Le Li

**Affiliations:** Department of Rehabilitation Medicine, Guangdong Engineering and Technology Research Center for Rehabilitation Medicine and Translation, The First Affiliated Hospital, Sun Yat-sen University, Guangzhou, China

**Keywords:** sit-to-stand, subacute stroke, kinematic, kinetic, rehabilitation

## Abstract

**Objective:**

The aim of this study was to detect the key changes during sit-to-stand (STS) movement cycle in hemiparetic stroke survivors using a five-phase kinematic and kinetic analysis.

**Methods:**

Twenty-five subacute stroke survivors and 17 age-matched healthy adults participated in this study. The kinematic and kinetic parameters during STS cycle were measured using three-dimensional motion analysis system with force plates. The five standard phases of STS cycle were identified by six timing transitional points.

**Results:**

Longer total time as well as larger changes were observed at the initial phase (phase I, 0.76 ± 0.62 VS 0.43 ± 0.09 s; *p* = 0.049) and at the end of hip and knee extension phase (phase IV, 0.93 ± 0.41 VS 0.63 ± 0.14 s; *p* = 0.008) in the stroke group than healthy group. Time to maximal knee joint moment was significantly delayed in the stroke group than in the control group (1.14 ± 1.06 VS 0.60 ± 0.09 s, *p* < 0.001). The maximal hip flexion was lower during the rising phase from seated position on the affected side in the stroke group than in the control group (84.22° ± 11.64°VS 94.11° ± 9.40°; *p* = 0.022). Ground reaction force was lower (4.61 ± 0.73 VS 5.85 ± 0.53 N, *p* < 0.001) in the affected side of the stroke group than in the control group. In addition, knee joint flexion was significantly lower at just-standing phase (T_4_) and at end point (T_5_) (5.12° ± 5.25° VS 8.21° ± 7.28°, *p* = 0.039; 0.03° ± 5.41° VS 3.07° ± 6.71°, *p* = 0.042) on the affected side than the unaffected side. Crucial decrease of knee joint moment at abrupt transitory (T_2_) and the maximal moment was also observed on the affected side in comparison with the unaffected side (0.39 ± 0.29 VS 0.77 ± 0.25 Nm/kg, *p* < 0.001; 0.42 ± 0.38 VS 0.82 ± 0.24 Nm/kg, *p* < 0.001).

**Conclusion:**

The findings of movement decomposition analysis provided useful information to clinical evaluation of STS performance, and may potentially contribute to the design of rehabilitation intervention program for optimum functional recovery of STS after stroke.

## Introduction

The ability to stand up from a seated position is very important in performing activities of daily living independently. It is also a prerequisite for gait (1, 2). The execution of sit-to-stand (STS) can be affected by several factors, including age, seat height, armrests, feet position, muscle strength, and balance ability (3–6). Stroke survivors suffer from impaired mobility and walking ability (7, 8) and these common symptoms contribute to STS disability that further confines their activities of daily life (9). Stroke survivors are prone to fall during STS because of the reduced ability of standing up from a chair (10). The severity of fear of falling was shown to be correlated with Timed up and Go test (11). Previous study revealed that 37.2% of falls in stroke survivors occurred while changing position from STS (12). The standardized evaluation on effectiveness of intervention to improve STS performance is still insufficient. There is also divergence on the clinical efficacy of standard beside repetitive practice of STS in rehabilitation clinic (9). Thus, the ability of rising from a chair remains difficult to recover after stroke (13). It is necessary to have an improved understanding on STS characteristics during the action implementation to improve training task performance and to decrease the fall rate in hemiparetic stroke survivors (14).

Sit-to-stand movement is the bridge between static position to dynamic body activity from the biomechanical view and defined as a transitional movement to the upright posture (15). The dynamic of STS is usually described using kinematic and kinetic variables. STS is generally assumed as a symmetrical activity of the lower extremity in the sagittal plane in healthy individuals (15–17). Previous researchers demonstrated that the influence of standing up velocity, center of pressure sway, and muscle activation pattern were related to the functional capability (18, 19). Chou and co-workers used a 3D motion analysis system combined with force plate to investigate the relationship between STS and gait parameters. The results indicated that a shorter duration of standing up or less vertical force difference on body weight distribution was associated with better gait performance (20). However, detailed phase analysis in STS is still limited which affects the further understanding of the specific motion deficits in stroke survivors during STS.

Previously, some authors studied the phases of habit development during STS activity from a biomechanical aspect and used it for optimization analysis (16, 21). The kinematic and kinetic data suggested that STS movement cycle could be divided into four or five phases (22–24). Galli and co-workers found prolonged STS in the ascending phase and different vertical forces in people after stroke in comparison with healthy controls (24). However, which specific phases are particularly crucial during the STS motion remains unclear. Previous studies on STS motion reported a wide variety of testing protocols, including difference in seat height, initial joint angle, and foot placement which led to different kinematic and kinetic results and conclusions (16, 21–24). In addition, the kinetic and kinematic characteristics of the timing and transitional points during STS cycle were seldom explored. Quantitative data such as movement duration, joint angle, and moment of timing and phases reference during STS of hemiparetic subjects are scarce, especially in stoke patients at subacute stage. Detailed motion analysis that depends on movement decomposition may identify typical pattern of change during STS after stroke. This would then contribute to the design of subject-specific training program based on the timing point and phase data for stroke survivors to regain the ability to perform STS task.

Therefore, this study aimed to explore the kinematic and kinetic characteristics of STS based on phases and transitional points analysis in bilateral lower limbs of subacute stroke survivors, and to compare the characteristic changes with healthy adults. The results of the current study would contribute to the knowledge of movement characteristics in people after stroke during STS and assist the development or evaluation of rehabilitation intervention of this specific motor task.

## Materials and Methods

### Subjects

Twenty-five subjects (17 male and 8 female, age from 43 to 77 years old) with subacute stroke (from 14 to 85 days after stroke) were recruited for this study. The characteristics of the stroke survivors were summarized in Table 1. Patients were selected according to the following inclusion criteria: (1) the occurrence of a first stroke with unilateral hemiparesis lesions confirmed by magnetic resonance imaging or computed tomography; (2) no more than 3 months after stroke; (3) ability to stand up and sit down independently more than six times from a standard chair; (4) abnormal 10-m walk time according to age. Abnormal 10-m walk time was defined as: (i) age <60 = total completion time of over 10 s or slower than 1 m/s, (ii) age 60–69 = over 12.5 s or slower than 0.8 m/s, (iii) age ≥70 = over16.6 s or slower than (0.6 m/s) (25); and (5) adequate mental and auditory capacity to follow oral commands (Mini mental state examination score ≥27).

**Table 1:** Anthropometric characteristics of the subjects.

	Patients Mean (SD)	Healthy subjects Mean (SD)	*p*-Value
Number of subjects	25	17	
Sex (male:female)	17:8	11:6	1.000
Age (years)	58.24 (10.46)	57.53 (6.63)	0.792
Days of post-injury	43.56 (23.65)	–	
Height (mm)	1,646 (73.43)	1,617 (62.13)	0.178
Body weight (kg)	66.24 (11.1)	64.45 (9.38)	0.858
Leg length (mm)	843.32 (42.21)	829.12 (35.54)	0.337

Seventeen healthy controls matched for age, weight, leg length, and gender participated in this study to provide the reference data for STS movement. All subjects had no history of back pain, joints pain, and no diagnosed lower limbs musculoskeletal disorders within 6 months prior to data collection. This study was approved by the Human Subjects Ethics Sub-committee of the First Affiliated Hospital, Sun Yat-sen University, China (ethic number [2014]0.88). The study was conducted in accordance to the Declaration of Helsinki. All subjects provided informed written consent prior to enrollment.

### Instrumentation

Vicon Motion Analysis System (VICON MX13, VICON Peak, Oxford, UK) was used for motion analysis. The locations of passive reflective markers taped to the skin overlying bony landmarks of the pelvis and lower limbs were: (1) the sacrum at the level of the posterior superior iliac spines; (2) anterior superior iliac spines; (3) lower lateral one-third and halfway points on the thighs; (4) lateral epicondyle of knees; (5) lower lateral one-third and halfway points of the shanks; (6) lateral malleolus; (7) the second metatarsal head; (8) the calcaneus at the same height as the second metatarsal head and; and (9) T10. Trajectories of reflective markers were recorded by six wall mounted infrared cameras. Two force plates (OR6-7, AMTI Inc., Watertown, MA, USA) embedded in the floor were used to record ground reaction force (GRF). Kinematic and kinetic data were captured using Vicon Nexus (version 1.7.1) and Plug-in-Gait. The data from VICON cameras were sampled at 100 Hz and the data from force plates were sampled at 1,000 Hz, simultaneously. The data from one frame of cameras parallel with 10 frames of force plate. Temporal, kinematic, and kinetic parameters were processed and analyzed using Polygon (version 3.5.1). A height-adjustable chair without back or armrests was used in the experiment.

### Experiment and Tasks

**Figure 1:**
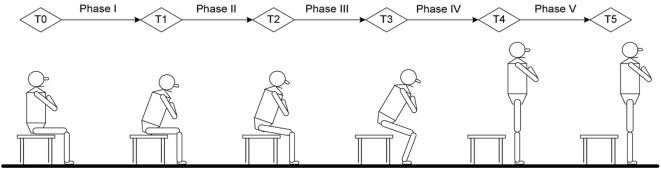
Time point and phase definition in current study: T0, initial point of trunk flexion; T1, point of maximal hip flexion; T2, point of abrupt transitory knee extension; T3, point of maximal ankle dorsiflexion; T4, point of just standing with full extension of hip and knee; T5, end of sit-to-stand (stable standing). Phase I, forward transfer of trunk; Phase II, hip lifting off the chair and maximal hip flexion; Phase III, transitory knee extension point to maximal ankle dorsiflexion; Phase IV, maximal ankle dorsiflexion to point of just standing up in nearly full extension of the knee and hip; Phase V, stable standing.

All of the subjects sat on the chair with seat height adjusted to keep the hip and knee joints as closed to 90°as possible. Both feet were placed on the force plates at neutral position and shoulder width apart. Arms were crossed on the chest with head facing forwards at a horizontal direction (Figure 1). For a complete movement cycle, subject was asked to stand up, remained stood up for 10 s, and then sat down. This was repeated for 10 cycles. Only the data from STS were analyzed. Subjects had a break of 5 s in between each complete cycle. The subjects were asked to keep their trunks and heads at upright position and to remain stationary as much as possible prior to STS task. They then started to rise from a seated position after the oral cue was heard. The start of STS was defined as the point where trunk flexion first occurred and was marked by the forward movement of T10 marker (22). The endpoint of stable standing was defined as the stoppage of the trunk and lower-limb markers (24). The transitional points of STS action were measured from the data on one force plate and angular movement. The data of the right and left lower limbs were collected by two force plates and markers trajectories. The task was performed at natural speed of the subjects’ habit and ability. All subjects were asked to keep both heels in contact with the force plates without moving their feet during STS movement and between cycles. A line marked at 50% of the thigh length was aligned with the anterior border of the seated chair. Kinetic, kinematic, and temporal variables were measured at sagittal plane during STS movement.

### Data Analyses and Statistics

The STS analysis was organized into phases that depend on kinematic variables, GRFs and momentum-transfer phase (13), STS movement has consistent pattern that can be divided into five phases and six transitional points based on biomechanical view and kinematic data (22, 24). The six transitional points are as follow: T_0_ = the initial point of trunk started to flex; T_1_ = the point of maximal hip flexion; T_2_ = the point where knee started to extend, which is also called abrupt transitory point; T_3_ = the point of maximal ankle dorsiflexion; T_4_ = just standing to full extension of hip and knee; T_5_ = end of STS (stable standing), also referred to as end point. The five phases of STS obtained from six timing points were defined as: (i) phase I—from T_0_ to before the hip left the seat (vector force begin to increase on force plate); (ii) phase II—from hip lifting off seat to T_1_; (iii) phase III—from T_2_ to T_3_; (iv) phase IV, from T_3_ to T_4_; and (v) phase V—from T_4_ to T_5_ (Figure 1).

Statistics analysis was performed using SPSS version 15.0. Descriptive statistics were computed for demographic characteristics and for all other parameters. Six trials with the smoothest and highest coincidence from the 10 recorded trials were included for data analysis according to reliability and validity of STS repetition (22, 24). The mean of the six STS cycles were computed for each subject. The Fisher’s exact test was used to compare the difference of gender ratio between the stroke group and the healthy group. Anthropometric data (age, body height and weight, leg length) for hemiparetic and healthy subjects were analyzed by independent samples *t*-test. Independent *t*-test was also used to assess the between group differences in timing phases and the time in point between stroke and healthy groups. The differences in joint angle, moment, and GRF of each transitional points at sagittal plane between the three groups (affected, unaffected, and healthy lower limbs) were assessed using one-way ANOVA. Statistical significant level was set as *p* < 0.05 (two tailed).

## Results

The stroke and healthy groups had no statistical significant difference in age and anthropometric parameters (Table 1).

**Figure 2:**
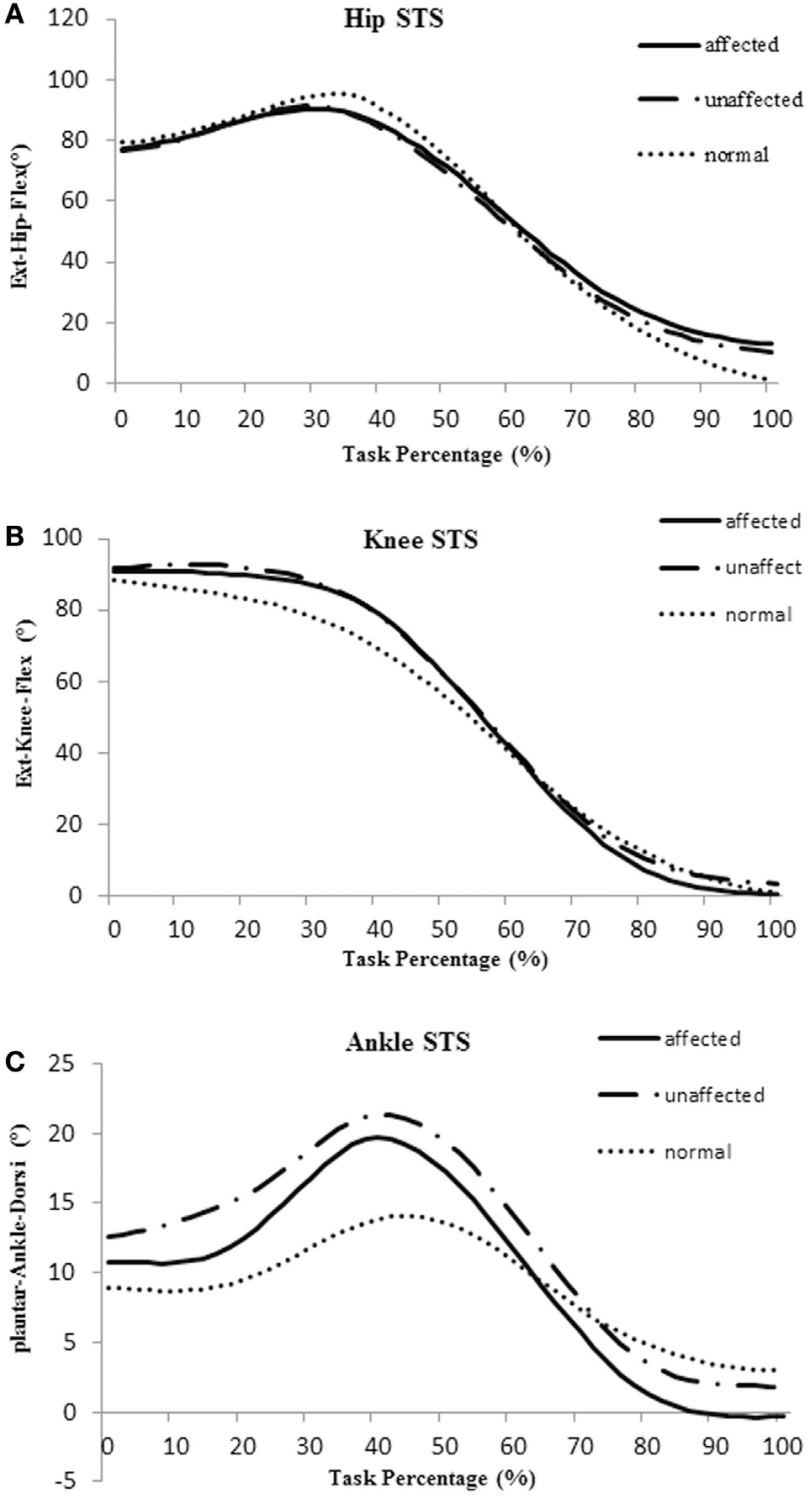
Averaged joint angle curves of hip **(A)**, knee **(B)**, and ankle **(C)** during sit-to-stand (STS) movement in the affected side (blue), unaffected side (red), and healthy control group (orange).

The total timing of a STS cycle was calculated from T_0_ to T_5_ and was converted to 100%. Averaged joint angle curves of hip, knee, and ankle during STS movement in the affected, unaffected, and healthy group are shown in Figure 2.

**Figure 3:**
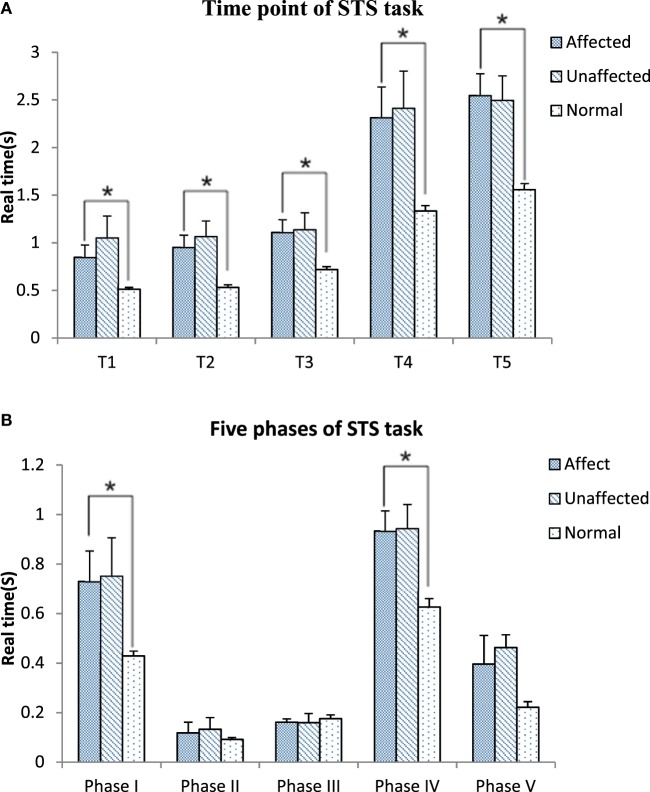
Time point **(A)** and five phases **(B)** comparisons among the affected, unaffected side of stroke and healthy controls during sit-to-stand (STS) task (*p < 0.05).

Figure 3 shows the timing point from T_1_ to T_5_ and the five phases in the stroke and healthy groups. All timing transitional points were significantly delayed in the stroke group when compared with the healthy group (*p* < 0.05). Phase I and phase IV were significantly lengthened in the stroke group when compared with the healthy group (0.76 ± 0.62 VS 0.43 ± 0.09 s, *p* = 0.049; 0.93 ± 0.41 VS 0.63 ± 0.14 s, *p* = 0.008, respectively) (Figure 3A). The angle at T_1_ point of maximal hip flexion was significantly lower in the stroke group (84.22° ± 11.64°) than the healthy group (94.11° ± 9.40°, *p* = 0.022), and maintained more flexion at end point (T_5_) of STS. The knee angle of affected limb in full extension point (T_4_) (5.12° ± 5.25°) was less flexed than the unaffected limb (8.21° ± 7.28°, *p* = 0.039). There was significantly smaller flexion angle on the affected limb than the unaffected limb at the end point (T_5_) of STS cycle for the stroke group (0.03° ± 5.41° VS 3.07° ± 6.71°, *p* = 0.042) (Figure 3B).

**Figure 4:**
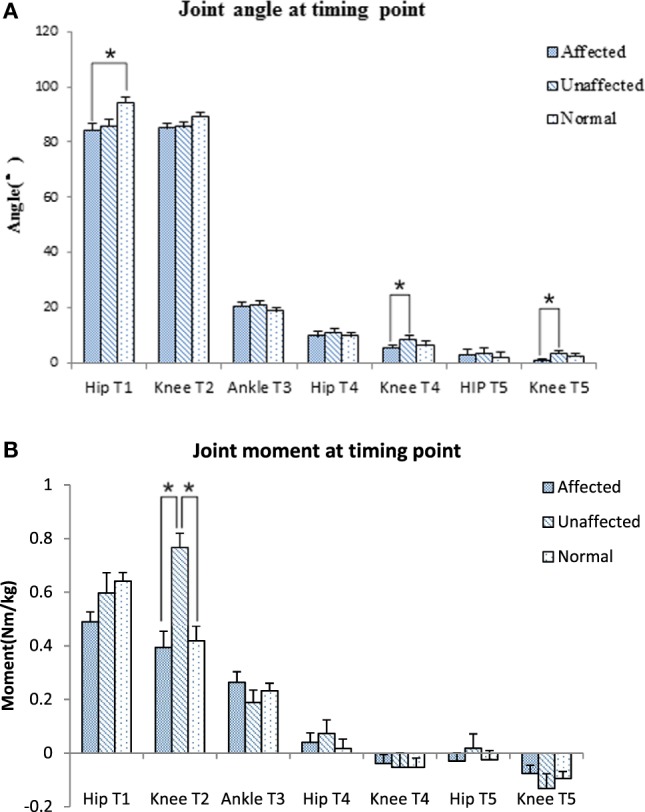
Joint angle **(A)** and joint moment **(B)** at timing point comparison among the affected, unaffected side of stroke, and healthy controls (*p < 0.05).

The knee joint moment was significantly lower in the affected limb than unaffected limb at abrupt transitory point (T_2_) (0.39 ± 0.29 VS 0.77 ± 0.25 Nm/kg, *p* < 0.001). The unaffected knee moment of the stroke group increased significantly compared with the healthy group at T_2_ point (0.77 ± 0.25 VS 0.42 ± 0.22 Nm/kg, *p* = 0.006) (Figure 4B). GRF decreased significantly at affected lower limb (4.61 ± 0.73 N) in comparison with the unaffected lower limb (6.69 ± 0.86 N) and healthy lower limb (5.85 ± 0.53 N, *p* < 0.001, respectively).

**Figure 5:**
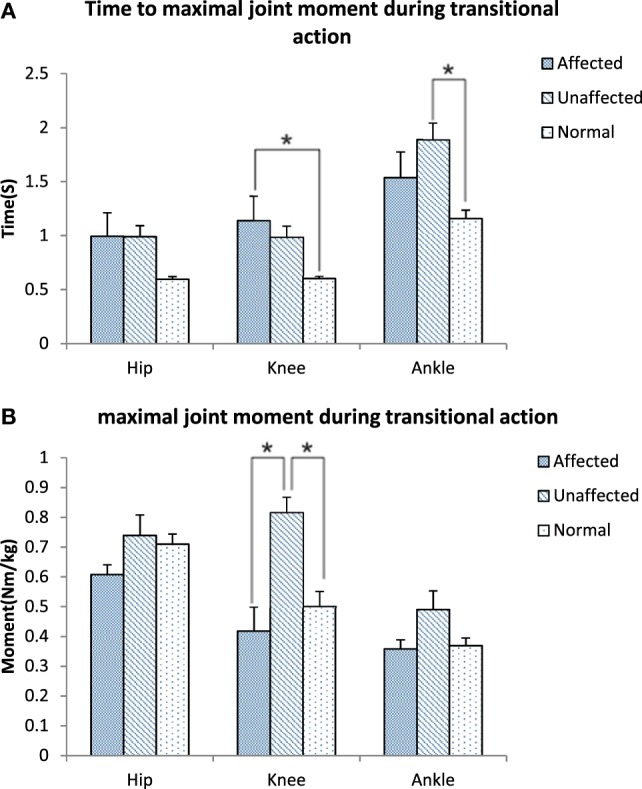
Time to maximal joint moment **(A)** and Maximal joint moment **(B)** during transitional action among the affected, unaffected side of stroke, and healthy controls (*p < 0.05).

The maximum knee joint moment was significantly lower on the affected limb than the unaffected limb in the stroke group (0.42 ± 0.38 VS 0.82 ± 0.24 Nm/kg, *p* < 0.001, Figure 5A). Time to maximal knee joint moment was significantly delayed in the stroke group when compared with the healthy group (1.14 ± 1.06 VS 0.60 ± 0.09 s, *p* < 0.001). Time to maximal ankle joint moment was significantly delayed in the unaffected limb of the stroke group when compared with the healthy group (1.89 ± 0.72 VS 1.16 ± 0.31 s, *p* = 0.027, Figure 5B).

## Discussion

This study used 3D motion analysis to explore the phases and transitional points of kinematic and kinetic differences of lower limbs during STS motion in subacute stroke survivors and compared those with age-matched healthy adults.

All of the transitional points were significantly delayed and the total time of STS task was significantly longer in the subacute stroke group when compared with healthy adults (Figure 3A). The results of the motion analysis indicated that the increase in total STS time was mostly related to the delay in phase I and phase IV (Figure 3B), where the majority of the time was spent on trunk flexion before the hip left the seat, and before hip and knee joints reached full extension. The increased time observed during phase I means that slow velocity of standing up from a seated position derives from the initial stage. Reduced velocity is proportionate to the disability of standing up previously reported in literature (18). Our findings were in line with Galli and co-workers who also found hemiplegic adults had a prolonged initial phase of STS (24). These changes might be related to poor trunk control and weak muscle strength (13, 26, 27). The increased total time of the STS task caused by lower speed indicates rising from a seated position is a challenging activity for subacute stroke survivors. It is recommended to incorporate the training of lean forward speed at the early stage of rehabilitation. In addition, the time point of hip lifting off the seat (the beginning of phase II) previously reported was notably earlier than the T_1_ point observed in our study. This led to a negative data of phase II in some subacute stroke survivors. This reverse strategy may also influence the stability of posture control (22) that contributes to increase risk of falling (10). Therefore, findings of this study suggest that trunk forward flexion and hip flexion should be particularly trained in people after stroke to enable a higher speed to shorten phase I of STS and keep hip joint fully flexed at T_1_.

The body of literature suggested that hip flexion angle contributed to rising from a seated position in healthy subjects (28). The results of this study showed that people with subacute stroke decreased maximal hip flexion angle during T_1_ when compared with healthy adults before standing up from a seated position. This indicated the hip left the seat before getting sufficient flexion (Figures 2A and 4A). This partly explained that subacute stroke survivors might encounter difficulty to stand and easy to fall when lifting from a seated position. An interesting finding from our study was that the affected knee joint had close to full extension to maintain stability at upright position (T5) and the unaffected knee joint maintained a slight flexion to maintain postural stability (Figures 2B and 4A). In contrast, hip joint remained slightly flexed at end point (T5). The inability to achieve full hip extension and knee extension in lock position with stroke survivors might be related to the weakness of the gluteus maximus and quadriceps femoris (13) muscles. The higher ankle anterior–posterior swung between dorsiflexion and plantar flexion in the stroke group (Figure 2C) might be one of the factors that influenced the balance function of people after stroke (29).

It is important to detect the moment in joint angles when rising from a seated position, which requires larger hip flexion and knee extension than walking or climbing (3). Our results showed that the knee joint moment at the point of abrupt transitory extension (T2) (Figure 4B) and GRF were significantly lower on the affected side when compared with the unaffected side and control subjects. In addition, our study also found the maximal hip flexion moment was synchronous with maximal knee extension moment on the unaffected side of the stroke group and on the healthy group, with the timing point of maximum moment of knee joint occurred after extension began on the affected limb (T2) (Figure 5B). These findings revealed that difficulties in rising from seated position might be related to weakness of lower extremity muscle strength and abnormal moment timing point. Subacute stroke subjects depend much more on the unaffected limb while implementing the STS movement, especially when knee extension started (30). Therefore, it might need to increase knee extensors strength in subacute stroke survivors, especially at initial extension position to maintain symmetry of STS movement pattern. Our study showed an unequal GRF between affected and unaffected side, while the total GRF of the stroke group were similar with healthy adults. Impairments in muscles strength, postural control and balance were factors previously shown that contributed to asymmetrical weight-bearing in patients with stroke (20, 31, 32). Subacute stroke survivors put less weight on the affected limb and increased the load of the unaffected limb. Similar results were reported in previous studies (33, 34). All of these findings suggested that balance and weight-bearing training were needed for people with stroke to improve their motor function and posture control to regain ability of STS.

People with stroke have difficulties to rise from a seated position. This ability is not easily recovered due to muscle weakness, impaired motor control, and balance function. In our study, the results would assist the development of a targeted rehabilitation interventions to improve STS ability for people after stroke. The training of trunk leaning forward can begin as early as possible while the person after stroke can sit up, and focus on increasing the velocity and amplitude of trunk and hip joint flexion. The muscles strength of gluteus maximus and quadriceps femoris must be reinforced by exercise, and should be trained at early stage of bed rest. In addition, by means of designing exercises at bed rest stage and exercise that mimic STS motion to increase GRF at affected limb. Therefore, these results may give a cue and guidance to improve STS performance.

This study has several limitations which limits the interpretation and generalizability of the data. First, we only recorded the motion data and GRF during STS. We did not have electromyography recording of the lower limb muscles nor the objective muscle strength data from dynamometer. Further combination of kinematic, electrophysiological signals and muscle strength measurements could increase understanding on the mechanisms of motor control changes during STS following stroke. This would lead to a better understanding of the alteration of movement sequence. Second, we focused on lower limb kinematic and kinetics characteristics and did not consider movement of the upper body such as the neck and shoulders. This prevented the detailed analysis of whole-body posture comparison and evaluation between healthy control and people after stroke during STS.

## Conclusion

The total time of STS task was significantly longer in people with subacute stroke when compared with the healthy controls. Knee moment, abnormal timing point, and GRF on the affected side during STS task were reduced. The observed kinematic and kinetic changes in subacute stroke survivors during STS provide suggestions to the clinical intervention of improvement of STS motor performance.

## Ethics Statement

The experiment was approved by the Human Subjects Ethics Sub-committee of the First Affiliated Hospital, Sun Yat-sen University, China (ethic number [2014]88).

## Author Contributions

YM, XW, and JZ analyzed the data, interpreted the results, and drafted the manuscript. YM, WL, JZ, MD, ZX, LC, and RB conducted the experiment and collected the data. XW contributed to the revision of MS and the answers to the comments from the reviewers and editor. DH and LL designed the study and performed all stages of the study including data collection, analysis, interpretation, and substantial revision of the manuscript. All the authors approved the final version of the manuscript.

## Conflict of Interest Statement

The authors declare that the research was conducted in the absence of any commercial or financial relationships that could be construed as a potential conflict of interest.
